# Connexin mRNA distribution in adult mouse kidneys

**DOI:** 10.1007/s00424-021-02608-0

**Published:** 2021-08-07

**Authors:** Lisa Geis, Franz-Fabian Boudriot, Charlotte Wagner

**Affiliations:** 1 Department of Nephrology, University Hospital Regensburg, Regensburg, Germany; 2 Institute of Physiology, University of Regensburg, Regensburg, Germany

**Keywords:** Connexins, Gap junctions, Kidney, mRNA, RNAscope

## Abstract

Kidneys are thought to express eight different connexin isoforms (i.e., Cx 26, 30, 32, 37, 40, 43, 45, and 46), which form either hemichannels or gap junctions serving to intercellular communication and functional synchronization. Proper function of connexins has already been shown to be crucial for regulation of renal hemodynamics and renin secretion, and there is also growing evidence for connexins to play a role in pathologic conditions such as renal fibrosis or diabetic nephropathy. Therefore, exact intrarenal localization of the different connexin isoforms gains particular interest. Until now intrarenal expression of connexins has mainly been examined by immunohistochemistry, which in part generated conflicting results depending on antibodies and fixation protocols used. In this work, we used fluorescent RNAscope as an alternative technical approach to localize renal connexin mRNAs in healthy mouse kidneys. Addition of RNAscope probes for cell type specific mRNAs was used to assign connexin mRNA signals to specific cell types. We hereby found Cx26 mRNA strongly expressed in proximal tubules, Cx30 mRNA was selectively detected in the urothelium, and Cx32 mRNA was found in proximal tubules and to a lesser extent also in collecting ducts. Cx37 mRNA was mainly associated with vascular endothelium, Cx40 mRNA was largely found in glomerular mesangial and less in vascular endothelial cells, Cx43 mRNA was sparsely expressed by interstitial cells of all kidney zones, and Cx45 mRNA was predominantly found in smooth muscle cell layers of both blood vessels and ureter as well as in mesangial and interstitial (fibroblastic) cells. Cx46 mRNA could not be detected. In summary our results essentially confirm previous data on connexin expression in the renal vasculature and in glomeruli. In addition, they demonstrate strong connexin gene expression in proximal tubules, and they suggest significant connexin expression in resident tubulointerstitial cells.

**Supplementary Information:**

The online version contains supplementary material available at 10.1007/s00424-021-02608-0.

## Introduction

Proper kidney function depends on multiple self-regulating (feedback) mechanisms for which signaling between different cell types is mandatory. Although being subject of continuous research, exact intercellular coordination is not completely understood.

Since gap junctions and connexins (Cx) as their smallest subunits have been assigned to several different renal cell types, they are supposed to play a role in intercellular communication. Lack or dysfunction of connexins has been reported to result in interference with signaling of the juxtaglomerular apparatus [1, 2], regulation of renin secretion [3–5], or renal hemodynamics [6], while mutation or lack of tubular connexins have not been reported with special renal phenotypes or alterations of hemodynamic behavior yet [7].

Gap junctions provide intercellular communication and functional synchronization by mediating rapid electrical and chemical signaling between neighbored cells [8, 9]. They are formed by two opposite hemichannels, also called connexons. Building a central aqueous pore, a connexon consists of six connexins, which determine the electrical and biochemical properties of the connexon respectively the whole gap junction. Man and mouse express 21 and 20 different connexin proteins, respectively, which show a high degree of interspecies similarity with regard to amino acid sequences and organ distribution [8, 9].

The formation of gap junctions has meanwhile been reported for several cell types in the kidney including certain but not all vascular, glomerular, and tubular cells [6, 10]. Regarding the latter, classical gap junctions have only been found in the proximal tubule [11, 12]. Meanwhile it is also known that hemichannels itself can fulfil signaling functions such as mediation of ATP release from cells into the extracellular space and initiating purinergic signaling to adjacent cells [8, 9]. Indeed accumulating evidence suggest tubular and JGA connexin hemichannels play a functional role in kidney homeostasis [13–16].

In addition to the changes in kidney function resulting from connexin dysfunction, also changes of connexin expression have been reported for diseased kidneys [17–21]. However, in this context, it remains still unclear, whether altered connexin expression is a result or a cause of kidney disease.

Human and mouse kidneys have been reported to express eight connexin isoforms, i.e., Cx 26, 30, 32, 37, 40, 43, 45, and 46 [10].

While the overall gene expression of connexins in the kidney could reliably be determined by measurements of whole kidney mRNA abundance by real time PCR, this method does not allow to assign connexins to exact cellular structures. Immunohistochemical localization of connexin proteins is sensitive to artefacts, because results critically depend on fixation protocols and specificity of antibodies used.

For better understanding of the functional roles of connexins in the healthy and diseased kidneys, it is mandatory to clearly localize the expression of the different connexin isoforms in the kidney. This study therefore aimed to obtain a comprehensive overview about the localization of connexin gene expression in mouse kidney. Instead of localizing connexin proteins, this work focused on localization of connexin mRNA as a prerequisite for connexin protein expression.

We therefore used RNAscope to detect expression of different connexins on mRNA level and to map it to cellular structures in the mouse kidney. RNAscope is a newer mRNA in situ hybridization method. Based on target RNA-specific oligonucleotide probes, which are hybridized in pairs to multiple RNA targets and then again hybridized to signal amplification molecules conjugated to a different fluorophore or enzyme, RNAscope allows detection of different mRNA within a histological context.

## Materials and methods

### Animals

Experiments were conducted on 14- to 16-week-old male C57 Bl/6 mice purchased from Jackson Laboratory, USA. Animals were maintained on standard rodent chow (0.6% NaCl; Ssniff, Soest, Germany) with free access to tap water.

Mice were anesthetized with ketamine-xylazine and perfused retrogradely through the abdominal aorta at a flow rate of 13.3 mL/min using 40 mL PBS followed by 40mL of 10% neutral-buffered formalin solution. The kidneys were removed, dehydrated in an ethanol series, and embedded in paraffin. Tissues were cut into sections of 5 mm. All animal experiments were performed according to the Guidelines for the Care and Use of Laboratory Animals published by the US National Institutes of Health and approved by the local ethics committee.

### In situ hybridization via RNAscope®

Localization of mRNA expression was studied with the RNAscope® Multiplex Fluorescent v2 kit (Advanced Cell Diagnostics ACD, Hayward, CA, USA), according to the manufacturer’s instructions. The kidneys were perfusion-fixed with 10% neutral buffered formalin solution, dehydrated in an ethanol series and embedded in paraffin as mentioned above. Hybridization signals were detected on 5-μm tissue sections using the TSA® Plus fluorophores Cy3 and Cy5 (PerkinElmer, Waltham, Massachusetts). Slices were mounted with ProLong Gold Antifade Mountant (Thermo Fisher Scientific, Waltham, MA) and viewed with an Axio Observer.Z1 Microscope (Zeiss, Jena, Germany). Positive and negative controls were routinely enclosed. RNAscope® probes are listed in Table 1. Nuclei were counterstained with DAPI.

**Table 1:** Names of the targets and catalogue numbers of the probes used in this work.

RNAscope®-Probe	Cat no.
Mm-Cx26	518881
Mm-Cx30	458811
Mm-Cx32	554311
Mm-Cx37	588591
Mm-Cx40	518041 or c2 518041-c2
Mm-Cx43	486191 or c2 486191-c2
Mm-Cx45	538911
Mm-Cx46	876501
Mm-CD31	316721 or c2 316721-c2
Mm-CX3CR1	314221 or c2 314221 c2
Mm-megalin	425881-c2
Mm-nephrin	433571-c2
Mm-aquaporin 2	452411 or c3 452411-c3
Mm-PDGFRß	411381 or c2 411381-c2 or c3 411381-c3
Mm-Acta2	319531 or c2 319531-c2
2-plex Negative Control Probe	320751 bacterial

## Results

With the exception of Cx46 mRNA, all of the so far kidney-related connexins (i.e., Cx26, Cx30, Cx32, Cx37, Cx40, Cx43, and Cx45) were reproducibly detected by RNAscope**.** To assign connexin gene expression to cellular structures, we used cell type specific markers such as megalin mRNA for proximal tubules [22, 23]; aquaporin-2 mRNA for collecting duct cells [24, 25]; CD31 mRNA for endothelial cells [26]; α-SMA mRNA for smooth muscle cells [27]; PDGFRß mRNA for fibroblasts, pericytes, and mesangial cells [28]; and CX3CR1 mRNA for dendritic cells [29].

The description of the results follows subdivision of the kidney in three functional compartments:

### Cx26, Cx30, and Cx32 mRNAs are expressed in the tubular epithelial compartment

**Fig. 1:**
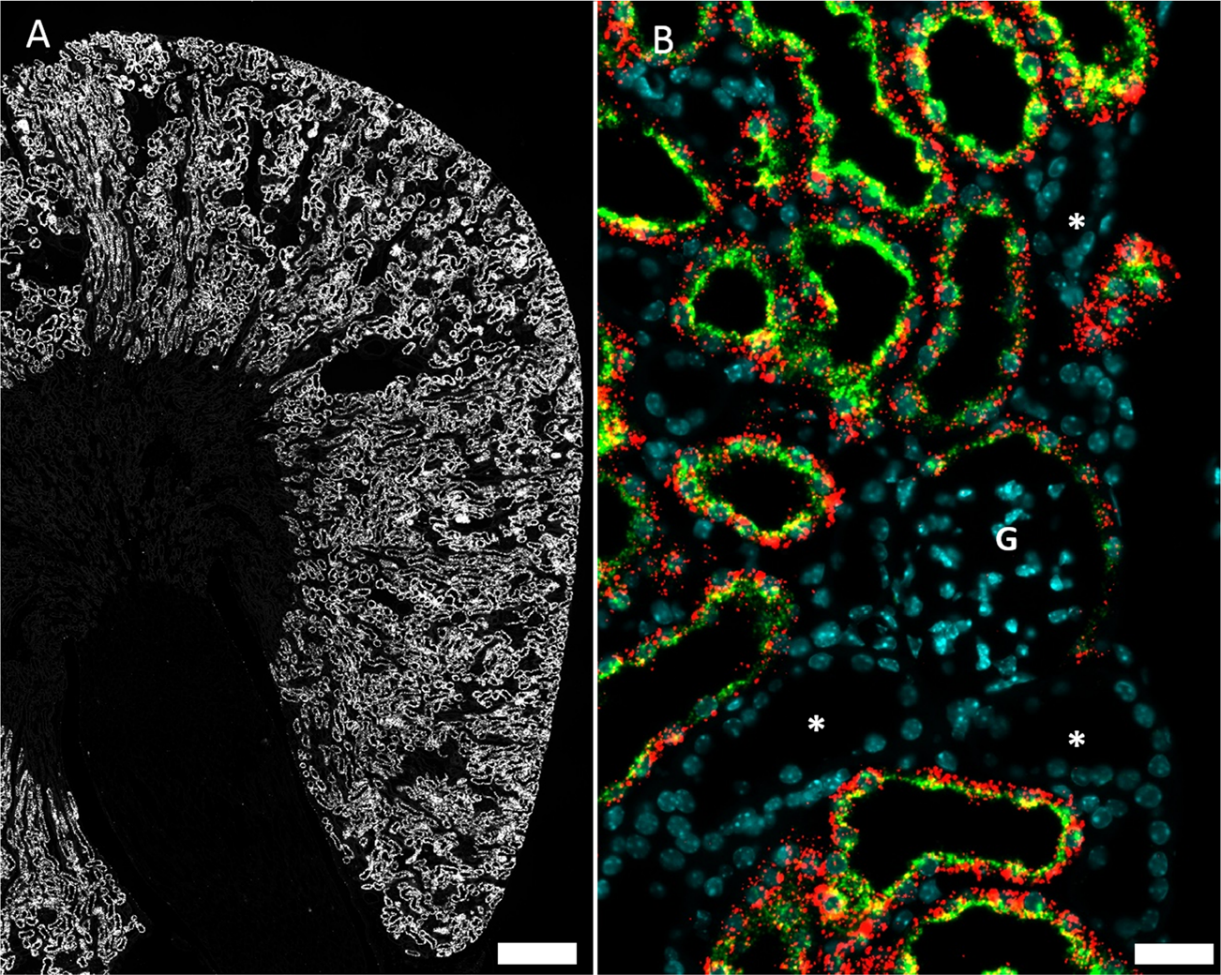
RNAscope for Cx26 mRNA in a normal mouse kidney. **A** Overview: Cx26 hybridization signals in white color. Signals are restricted to the kidney cortex and outer stripe of outer medulla; no signals are found in inner stripe of outer medulla or in inner medulla; size bar 500 μm. **B** Co-hybridization of Cx26 mRNA (red) with megalin mRNA (green) and nuclear DAPI staining (blue); size bar 20 μm. Consistent overlap of Cx26 mRNA and megalin mRNA. Glomeruli (G) and other segments of the renal tubule (asterisk) are negative for both Cx26 and megalin mRNA; size bar 30 μm

In whole kidney sections, the most and strongest mRNA signals were found for Cx26 mRNA and Cx32 mRNA. Cx26 mRNA was restricted to the kidney cortex and co-localized with megalin mRNA (Fig. 1) suggesting expression of Cx26 mRNA in all segments of the proximal tubule.

**Fig. 2:**
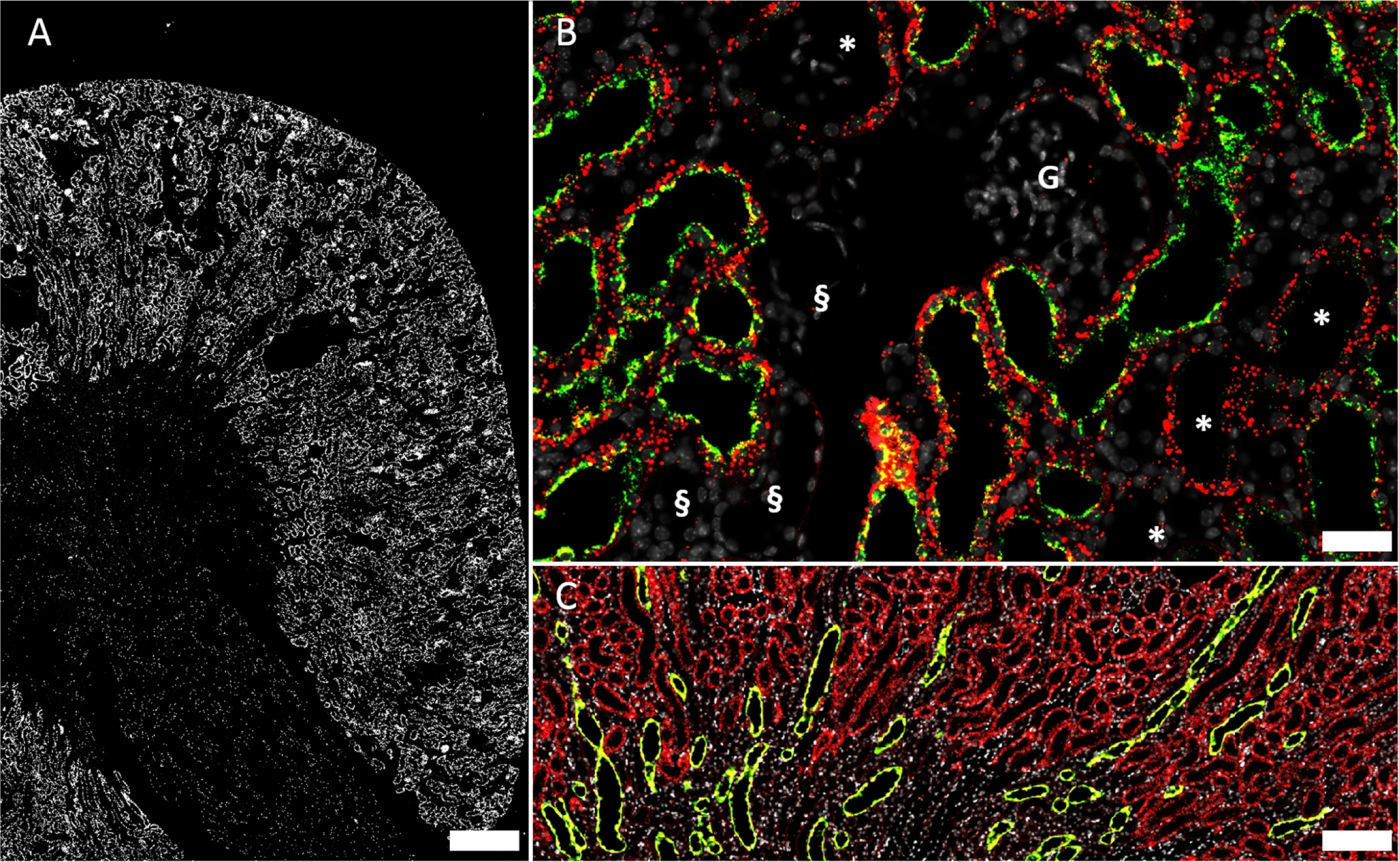
RNAscope for Cx32 mRNA in a normal mouse kidney. **A** Overview: hybridization signals in white. Strong signals are found in the kidney cortex, weaker signals are found in outer and inner medulla; size bar 500 μm. **B** Co-hybridization of Cx32 mRNA (red) with megalin mRNA (green) and nuclear DAPI staining (white). Picture shows sections of three different tubular segments as there are tubular cells with staining for both Cx32 mRNA and megalin mRNA as well as tubular cells with signal for Cx32 mRNA only (asterisk) and also tubular segments, which neither express Cx32mRNA nor megalin mRNA (§). Glomeruli (G) are negative for both Cx32 and megalin mRNA; size bar 30 μm. **C** Co-hybridization of Cx32 mRNA (red) with AQP2 mRNA (green) and nuclear DAPI staining (white). AQP2 mRNA expressing cells consistently also express Cx32 mRNA as indicated by yellow color merge. Expression of Cx32 mRNA beyond kidney cortex is restricted to AQP2 mRNA positive cells; size bar 100 μm

Cx32 mRNA was also strongly expressed in the kidney cortex and co-localized with megalin mRNA indicating expression by proximal tubules (Fig. 2A, B). In addition, Cx32mRNA was found—albeit at lower levels—in cells also expressing aquaporin 2 (AQP2) mRNA, suggesting expression by collecting duct cells in all kidney zones (Fig. 2C)

**Fig. 3:**
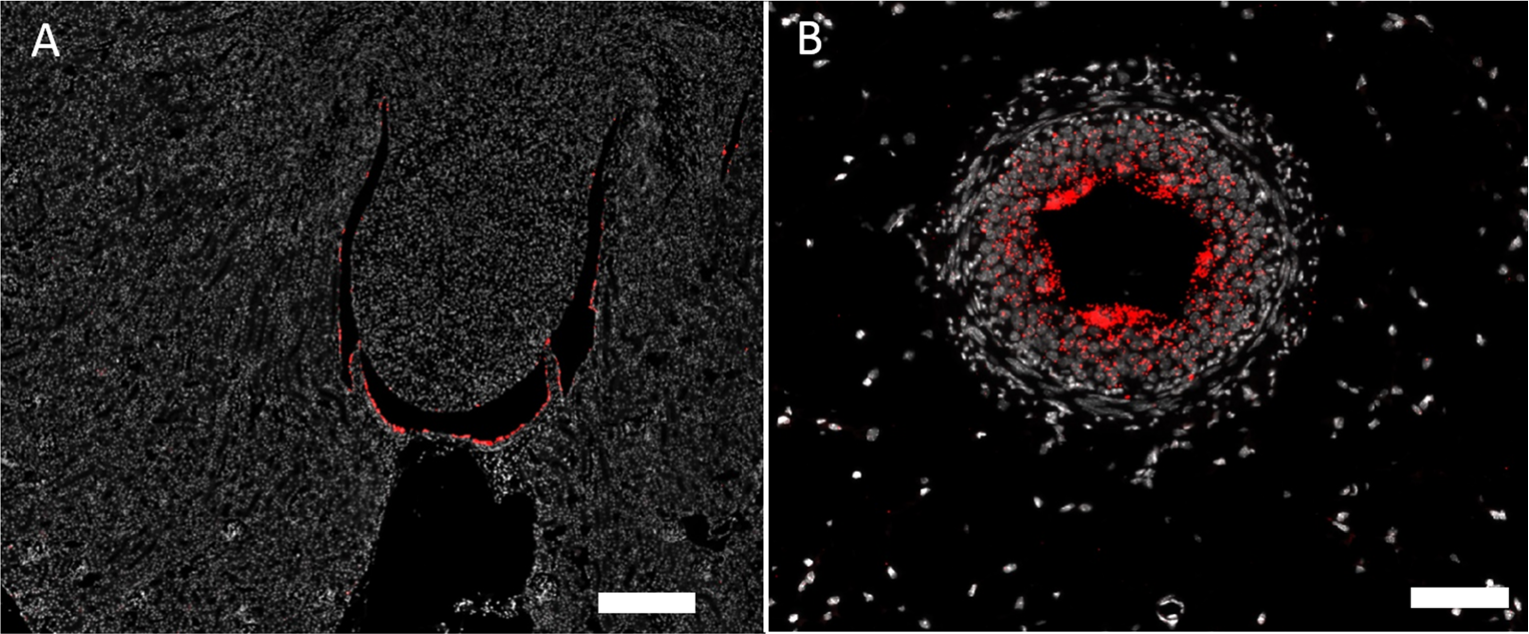
Cx30 mRNA in a normal mouse kidney. **A** Overview: cx30 mRNA hybridization signals in red and nuclear DAPI staining in white. Cx30 mRNA hybridization signals are restricted to cells lining the renal pelvis; size bar 200 μm. **B** Cross section of the ureter; hybridization signals in red color are found in the urothelium, nuclear DAPI staining in white; size bar 70 μm

Among the different connexin isoforms we examined, the expression of Cx30 mRNA appeared most restricted. Cx30 mRNA was only found in the thin layer of the urothelium of the renal pelvis (Fig. 3A). In addition, Cx30 mRNA was found in the urothelium of the ureter (Fig. 3B).

mRNA of Cx37, Cx40, Cx43, and Cx45 could not be detected in tubular cells.

### Cx37, Cx40, and Cx45 mRNAs are expressed in the vascular-glomerular compartment

**Fig. 4:**
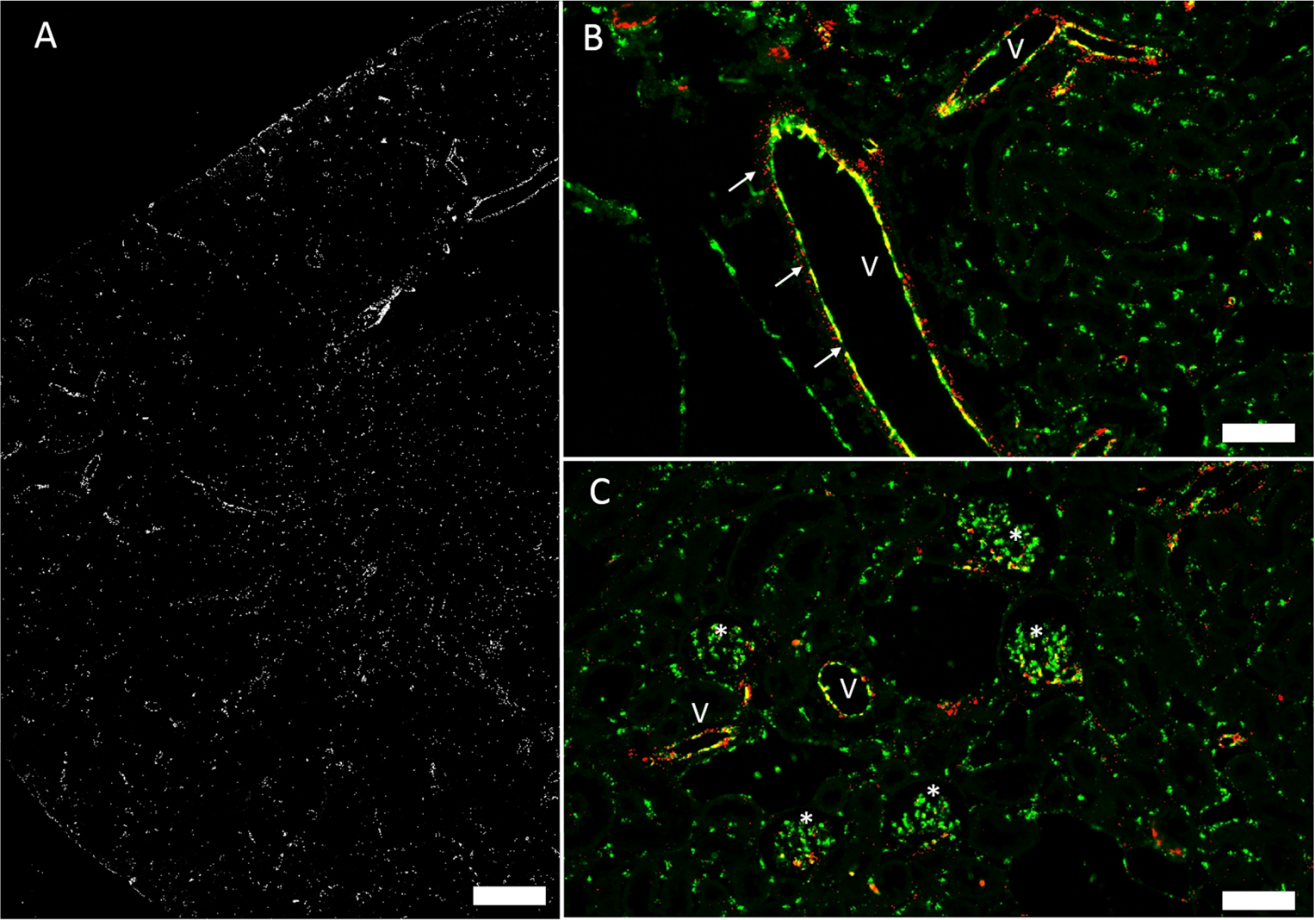
RNAscope for Cx37 mRNA in a normal mouse kidney. **A** Overview: Cx37 mRNA hybridization signals in white. Signals are found in all kidney zones but emphasized in vessels and in medullary rays; size bar 200 μm. **B** Co-hybridization of Cx37 mRNA (red) with CD31 mRNA (green). Clear co-expression of both mRNA types as indicated by yellow color fusion is found in blood vessels (v). In addition to endothelial cells, also some vascular cells of the smooth muscle cell layer express Cx37 mRNA (arrows); size bar 50 μm. **C** Co-hybridization of Cx37 mRNA (red) with CD31 mRNA (green). In glomeruli (asterisk) a subset of CD31 mRNA expressing cells additionally express Cx37 mRNA as indicated by yellow color merge. Interstitial dispersed CD31 mRNA expressing cells do not co-express Cx37 mRNA; size bar 50 μm.

Cx37 mRNA was readily detectable in blood vessels, glomeruli, and medullary rays (Fig. 4A). In vessels Cx37 mRNA signal merged with CD31 mRNA signal suggesting endothelial expression (Fig. 4B). In major intrarenal vessels, Cx37 mRNA further co-localized with α-SMA mRNA suggesting additional expression of Cx37 mRNA also by some vascular smooth muscle cells (suppl. Fig 1). In glomeruli Cx37 mRNA was found only in a subset of also CD31 mRNA expressing cells, which were localized around the vascular pole of the glomeruli (Fig. 4C). Apart from medullary rays, no significant expression of Cx37 mRNA was found in kidney interstitium (see the “Cx37 and Cx40 mRNAs are expressed in medullary rays; Cx43 and C45 mRNA are expressed by resident tubulointerstitial cells” section). We furthermore found no expression of Cx37 mRNA in tubular cells.

**Fig. 5:**
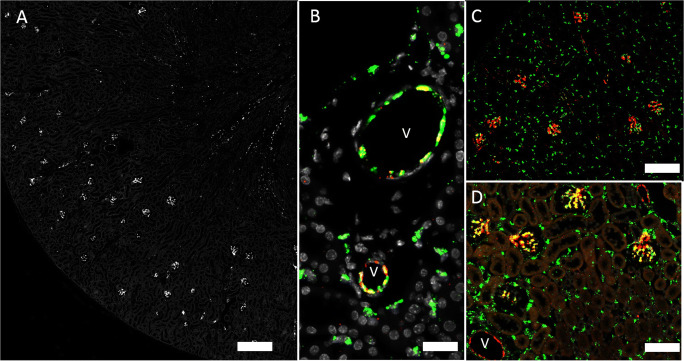
Cx40 mRNA in a normal mouse kidney. **A** Overview: hybridization signals in white. Signals are most prominent in glomeruli and in medullary rays; size bar 200 μm. **B** Co-hybridization of Cx40 mRNA (red) with CD31 mRNA (green), nuclear DAPI staining in white. Clear co-expression of both mRNAs as indicated by yellow color merge is found in blood vessel endothelium (v); size bar 30 μm. **C** Co-hybridization of Cx40 mRNA (red color) with CD31 mRNA (green color). Glomeruli show partial co-expression of Cx40 with CD31 as indicated by inconstant of yellow color merge; size bar 100 μm. **D** Co-hybridization of Cx40 mRNA (red) with PDGFRß mRNA (green). Clear evidence for glomerular co-expression of Cx40 mRNA with PDGFRß mRNA as indicated by yellow color merge; size bar 50 μm

In overview sections, Cx40 mRNA expression was found in blood vessels, glomeruli, and medullary rays (Fig. 5A). In vessels Cx40 mRNA co-localized with CD31 mRNA suggesting expression by vascular endothelial cells (Fig. 5B). Within glomeruli a hilar subset of CD31 mRNA expressing cells co-expressed Cx40 suggesting that intraglomerular endothelial cells at the glomerular hilum also express Cx40. There was strong co-expression of Cx40 mRNA and PDGFRß mRNA in glomeruli, suggesting expression of Cx40 mRNA by mesangial cells. Cx40 mRNA was also found in medullary rays (see the “Cx37 and Cx40 mRNAs are expressed in medullary rays; Cx43 and C45 mRNA are expressed by resident tubulointerstitial cells” section), but not in other interstitial cells.

**Fig. 6:**
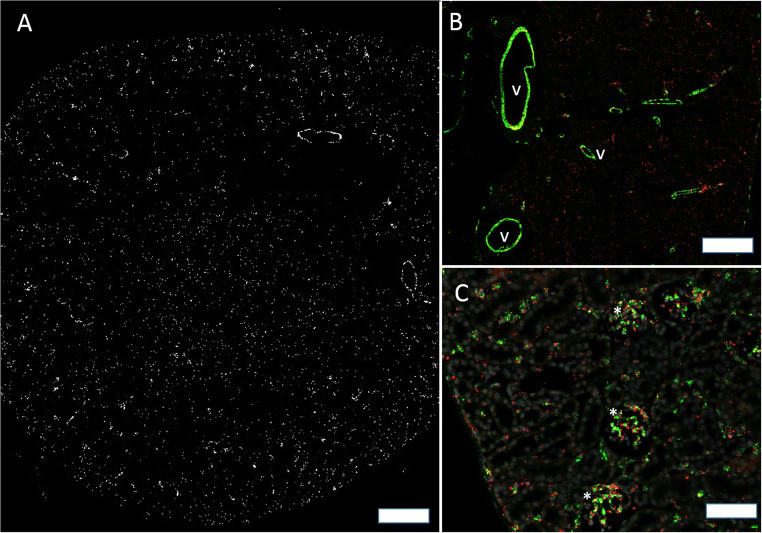
Cx45 mRNA in a normal mouse kidney. **A** Overview: hybridization signals in white. Signals are most prominent in vessels, glomeruli, and interstitial cells in all kidney zones; size bar 500 μm. **B** Co-hybridization of Cx45 mRNA (red) with α-SMA mRNA (green). Clear co-expression of both mRNAs as indicated by yellow color merge is found in blood vessels; size bar 200 μm. **C** Co-hybridization of Cx45 mRNA (red) with PDGFRß mRNA (green). Co-expression of Cx45 mRNA with PDGFRß mRNA in glomeruli (asterisk) and in interstitial cells is indicated by yellow color merge; size bar 100 μm

In overview sections, Cx45 mRNA was found in blood vessels and additionally diffusively distributed over all kidney zones (Fig. 6A). In vessels Cx45 co-localized with α-SMA mRNA suggesting expression of Cx45 mRNA in vascular smooth muscle cells (Fig. 6B). In glomeruli Cx45 mRNA co-localized with PDGFRß mRNA suggesting expression of Cx45 mRNA by mesangial cells (Fig. 6C). Furthermore, interstitial cells expressed Cx45 mRNA (see the “Cx37 and Cx40 mRNAs are expressed in medullary rays; Cx43 and C45 mRNA are expressed by resident tubulointerstitial cells” section). Cx45 mRNA was also found in the smooth muscle cells of the ureter (suppl. fig. 2).

**Fig. 7:**
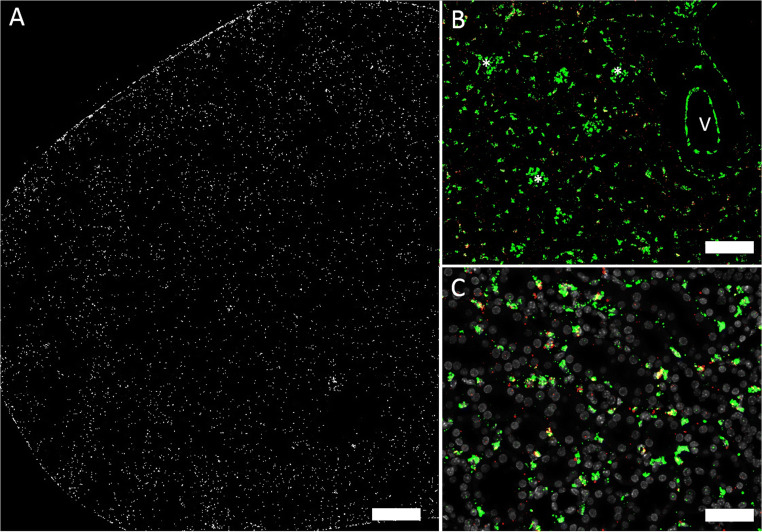
Cx43 mRNA in a normal mouse kidney. **A** Overview: Cx43 mRNA hybridization signals in white. Moderate signal is distributed over all kidney zones, a definite correlation to certain cell types is not possible; size bar 500 μm. **B** Co-hybridization of Cx43 mRNA (red) with CD31 mRNA (green). No co-expression of both mRNAs was found in blood vessels (v) or in glomeruli (asterisk); size bar 100 μm. **C** Co-hybridization of Cx43 mRNA (red) with CD31 mRNA (green), nuclear DAPI staining (white). Note some co-localization of Cx43 mRNA with CD31 mRNA in interstitial cells as indicated by the yellow color merge.

Compared with the distribution of the connexin isoforms described above, Cx43 mRNA showed the most diffuse expression (Fig. 7A). Cx43 mRNA could not clearly be mapped to blood vessels, glomeruli, or tubules (Fig. 7B, C). Instead Cx43 mRNA signal was mainly found in the tubulo-interstitium (see the “Cx37 and Cx40 mRNAs are expressed in medullary rays; Cx43 and C45 mRNA are expressed by resident tubulointerstitial cells” section). In view of the rather modest and more diffusively distributed hybridization signals obtained for Cx43 mRNA in the kidney, we performed RNAscope for Cx43 mRNA also on mouse heart tissue as a control. In heart sections, RNAscope for Cx43 revealed abundant hybridization signals in the myocardium suggesting that the weaker Cx43 mRNA signals in the kidney are not a technical artefact (suppl. fig. 3).

### Cx37 and Cx40 mRNAs are expressed in medullary rays; Cx43 and C45 mRNA are expressed by resident tubulointerstitial cells

Since connexin expression in the tubulo-interstitium has so far not been systematically examined, we also focused on connexin mRNA expression there. The renal interstitium mainly consists of three cell types, namely PDGFRß expressing fibroblasts and pericytes, CD31 expressing capillary endothelial cells, and CX3CR1 expressing dendritic cells.

**Fig. 8:**
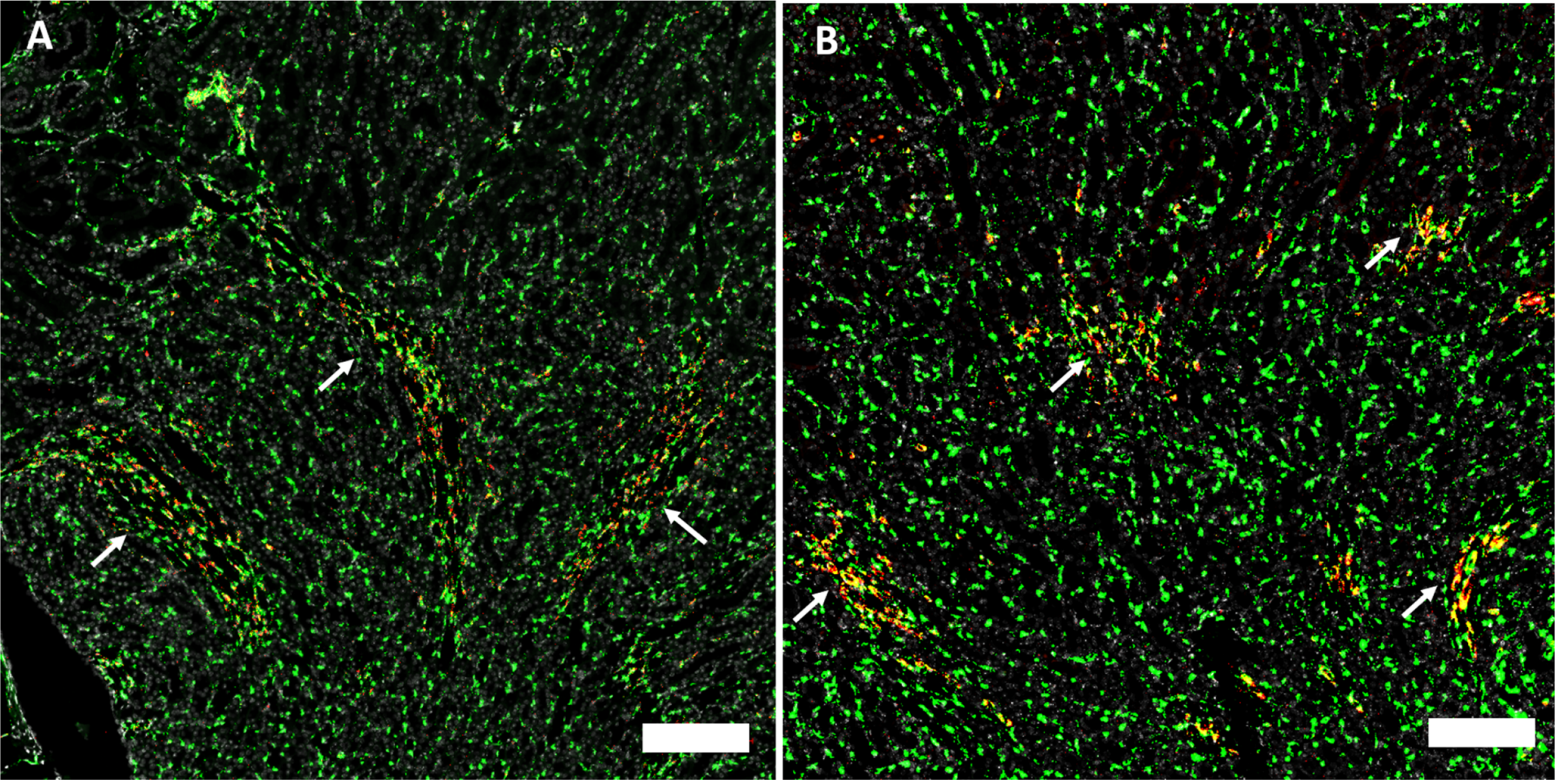
Connexin mRNAs in medullary rays. **A** Co-RNAscope for Cx37mRNA (red) and CD31 mRNA (green) on a normal mouse kidney section; arrows highlight medullary rays; size bar 200 μm. **B** Co-RNAscope for Cx40 RNA (red) and CD31 mRNA (green color) on a normal mouse kidney section; arrows highlight medullary rays; size bar 200 μm

The interstitial compartment expression of Cx37 mRNA as well as Cx40 mRNA was strongly associated with medullary rays, where both connexin mRNAs co-localized with CD31 mRNA suggesting expression by endothelial cells (Fig. 8). Beyond the expression in medullary rays, Cx37 mRNA and Cx40 mRNA were hardly detectable in the interstitium.

CD31 mRNA expression in interstitial cells beyond medullary rays showed partial overlap with Cx43 mRNA suggesting interstitial capillary endothelial cells might express Cx43 (suppl fig. 4). Since dendritic cells in other organs have been reported to express Cx43 [30–32], we examined, whether there was mRNA co-expression of Cx43 with dendritic cell marker CX3CR1. As shown in supplementary figure 5, we obtained no evidence for a significant overlap of Cx43 mRNA and CX3CR1 mRNA in the healthy mouse kidney. For mRNA of other connexin isoforms, we also found no co-expression with CX3CR1 mRNA (data not shown).

**Fig. 9:**
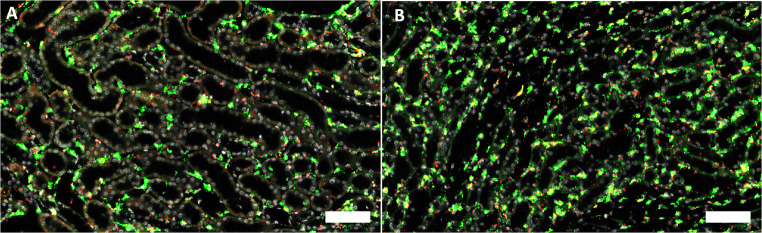
Co-RNAscope for CX45 (red) and PDGFRß (green) and nuclear DAPI staining (white) on a normal mouse kidney section. **A** Cortical area and **B** medullary area; size bars 100 μm. Note the overlap of Cx45 mRNA and PDGFRß mRNA expression as indicated by yellow color merge

Separate from medullary rays, Cx45 mRNA showed the strongest interstitial expression compared to all connexin isoforms we examined. Cx45 mRNA signal co-localized with PDGFRß mRNA suggesting expression of Cx45 by fibroblasts and pericytes (Fig. 9).

Table 2 provides a descriptive summarizing overview of the renal connexin mRNA distribution we found in this work:

**Table 2:** Connexin mRNA expression by different kidney cell types

	Cx26	Cx30	Cx32	Cx37	Cx40	Cx43	Cx45	Cx46
Proximal tubule	+++	-	+++	-	-	-	-	-
Thin limb loop of Henle	-	-	-	-	-	-	-	-
Thick limb loop of Henle	-	-	-	-	-	-	-	-
Distal tubule	-	-	-	-	-	-	-	-
Collecting duct	-	-	+(+)	-	-	-	-	-
Urothelium	-	++	-	-	-	-	-	-
Vascular endothelium	-	-	-	++	++	-	-	-
Vascular smooth muscle	-	-	-	(++)	-	-	++	-
Glomerular endothelium	-	-	-	+(+)	(+)	-	-	-
Glomerular mesangium	-	-	-	-	+++	-	++	-
Glomerular podocytes	-	-	-	-	-	-	-	-
Medullary ray endothelium	-	-	-	++	++	-	-	-
Interstitial capillary endothelium	-	-	-	-	-	(+)	-	-
Interstitial fibroblasts	-	-	-	-	-	-	++	-

+++ strong expression; ++ moderate expression; +weak expression; - no expression; (..) variable expression

## Discussion

This work was designed as a complementary technical approach study to localize connexin expression in the kidney.

Since so far connexin expression has mainly been localized by immunohistochemistry or reporter gene tracing, we will discuss our findings on connexin mRNA localization in comparison with published data on connexin protein immunohistochemistry.

Our findings on the localization of Cx26 and Cx32 mRNA in proximal tubules are in line with early and limited immunohistochemistry data obtained with mouse and rat kidney [33, 34]. The eye-catching basolateral expression of Cx26 mRNA, providing barely signaling overlap with megalin mRNA, needs to be mentioned. This phenomenon of so called subcellular mRNA localization has already been described [35]. One might assume that Cx26 and Cx32 form heterotypic gap junctions within the proximal tubule. The proximal tubule is the only segment of the nephron, for which gap junctions have been described so far [11, 36], albeit the functional role of these gap junctions has not yet been identified. Expression of Cx32 in collecting ducts as suggested by the existence of Cx32 mRNA in AQP2 mRNA positive cells has not yet been reported before. Since existence of gap junctions has not yet been described for collecting duct cells, the presence of Cx32 hemichannels is conceivable more likely. So far a hemichannel function in the distal nephron and collecting ducts has been suggested for Cx30 [15]. Our study, however, did not provide evidence for an expression of Cx30 mRNA in tubular structures, but we found Cx30 mRNA in the urothelium instead. We cannot exclude from our findings that Cx30 mRNA levels in tubular structures were below the detection limit of RNAscope but might be sufficient for functional relevant protein synthesis. Expression of connexins including Cx30 respectively existence of gap junctions in the urothelium has not been reported so far. Our data suggest that the expression of Cx30 extends from the renal pelvis along the ureter. Whether Cx30 form hemichannels or gap junctions in the urothelium as well as their possible functions need to be clarified. One could speculate that Cx30 connexins could contribute to mechanical signal propagation from the renal pelvis to the ureter, where the urothelium could couple via heterotypic Cx30/Cx45 gap junctions with ureter smooth muscle cells as reported by [37] and also found in this study. In contrast to a previous study [38], we could not find evidence for expression of Cx37 by tubular cells.

To date renal connexin distribution is best documented for the vascular-glomerular compartment. A number of studies have examined vascular connexin protein expression as well as their possible functional role [6, 10]. Our findings on the expression of Cx37 and Cx40 mRNAs in the vascular endothelium and of Cx45 mRNA in renovascular smooth cells are in accordance with the results of previous studies, which have localized Cx37 [39, 40] and Cx40 [40–42] by immunohistochemistry and Cx45 by gene reporter tracing [3, 37, 43]. In contrast to immunohistochemical studies, which related Cx43 to the vascular endothelium [39, 40, 42, 44], we could not detect Cx43 mRNA there. Since it has commonly been described that the overall abundance of Cx43 mRNA in healthy kidney is rather low [40, 41, 45], we cannot exclude that the level of Cx43 mRNA in the vascular endothelium was below the detection limit of the RNAscope technique, but was still high enough for significant protein expression. Comparing endothelial protein expression of Cx37, Cx40, and Cx43 by immunohistochemistry, lower expression of Cx43 is a common finding indeed [39, 40, 42].

In glomeruli we found most prominent expression for Cx40 mRNA, which almost exclusively matched mesangial cells, thereby confirming data obtained with immunohistochemistry [39–42]. Regarding Cx40 expression in glomerular endothelial cells, existing data is conflicting [40, 41]. In this work, we did find overlap of Cx40 mRNA with CD31 mRNA signals suggesting expression of Cx40 by glomerular endothelial cells, albeit co-signaling was inconstant. As co-expression of Cx40 and CD31 mRNA was mostly restricted to the vascular poles of the glomeruli, findings might be biased by kidney sectioning.

Our mRNA data also agree with previous reports about locally restricted glomerular expression of Cx37 [39, 40, 42, 46], which we would assign to a subset of endothelial cells localized at the vascular hilum rather than to mesangial cells, as there was overlap with expression of CD31 mRNA. But our results confirm a previous report [3], which describes the expression of Cx45 by mesangial cells.

In contrast to single studies on rat kidneys [47, 48], we could not obtain evidence for connexin gene expression in podocytes.

In the interstitium, we found strong expression of Cx37 and Cx40 mRNA matching CD31 expressing endothelial cells of medullary rays, what again confirms previous reports [39–41]. Interstitial cells beyond medullary rays showed expression of Cx43 or Cx45 mRNA. We did find Cx43 mRNA expression within CD31 positive capillary endothelial cells, which has been described before [39, 40, 42]. But remaining Cx43 mRNA positive interstitial cells neither expressed PDGFRß nor CX3CR1, therefore, neither fitting fibroblasts nor dendritic cells clearly. As described by others, Cx43 might probably be related to the endothelium of lymphatic vessels [44, 49]. Since multiple studies suggest a link between Cx43 expression and (pro-)fibrotic conditions of the kidney [17, 19, 50], even consider Cx43 to be a therapeutic target for therapy of renal fibrosis [17, 51], further investigation concerning exact localization and function of Cx43 in renal interstitium is needed.

A novel finding of our study is the expression of Cx45 mRNA by tubulointerstitial cells co-expressing PDGFRß. These interstitial fibroblast- and pericyte-like cells with their stellate shape form numerous contacts among each other but also with other neighbored cell types such as immune or tubular cells. The potential signaling pathways shared by tubulointerstitial cells have not yet been identified. The existence of Cx45 in interstitial cells could provide a basis for intercellular coupling or for purinergic signal release through hemichannels. Since we found no Cx45 mRNA expression by tubular cells, a potential coupling between interstitial cells and tubular cells would require formation of heterotypic gap junctions involving Cx45, Cx26, or Cx32. We did not obtain evidence for possible coupling of tubulointerstitial cells with resident immune cells, as we found no co-expression of any of the connexin isoform mRNA with CX3CR1 mRNA [29]. As PDGFRß expressing cells also play a crucial role in the development of renal fibrosis [52, 53], our finding of Cx45 mRNA in these cells is of special interest and also needs further investigation.

In summary, our data on localization of connexin mRNA in non-tubular cells corresponded well with available immunohistochemical findings. We could not confirm Cx43 expression on mRNA level as ubiquitous as described for Cx43 protein expression. In tubuli we could confirm expression of Cx26 and Cx32 mRNA, but not of Cx30 or Cx37. The very high abundance of Cx26 and Cx32 mRNA in proximal tubuli should be noted. Novel findings are the selective expression of Cx30 in the urothelium and of Cx45 in the tubulo-interstitium. Noticeable is the circumstance that many renal cell types feature expression of two different connexin isoforms (e.g., Cx26/32 in proximal tubules, Cx37/40 in endothelial cells, Cx40/45 in mesangial cells), thereby providing an option for formation of heterotypic gap junctions.

We are aware that this study has its limitations. As mentioned above, we cannot exclude that mRNA levels below the detection limit of the RNAscope technique can eventually still lead to protein synthesis of functional relevance and therefore explain divergent findings comparing mRNA with protein expression. Likewise, it is not possible to deduce relevant protein function from solely mRNA evidence. Correlation of mRNA and protein expression is stated to be poor in general, and there is data that Cx mRNA is not translated to protein automatically [45]. On the other hand, mRNA expression is still a prerequisite for protein expression, and transcription and translation are stated to be important control mechanisms of connexin expression [8], so detection of Cx mRNA should nevertheless be a useful information. In different kidney disease models, rise of Cx43 mRNA concentration was accompanied by increased protein expression [17, 54]. As existing data on renal connexin expression is still conflicting and results obtained with connexin staining protein antibodies are permanently questioned, the large congruency between our data and previous immunohistochemistry studies provides an important confirming aspect.

## Supplementary information

Figure 1 RNA scope showing co-localizition of both Cx37 mRNA (red) and a-SMA mRNA (green) in arterial smooth muscle layer; size bar 50 μm (PNG 1568 kb)

High Resolution Image (TIF 891 kb)

Figure 2 Co RNAscope for Cx45 mRNA (red) and α-SMA (green and nuclear DAPI staining (white) on a cross section of the ureter; size bar 50 (PNG 1952 kb)

High Resolution Image (TIF 1151 kb)

Figure 3 RNAscope for Cx43 mRNA (red) and nuclear staining (green) on a normal adult mouse heart section; size bar 100 μm; Note co-localization of Cx43 mRNA with nuclei as indicated by yellow color merge (PNG 3970 kb)

High Resolution Image (TIF 1864 kb)

Figure 4 Cx43 mRNA in tubulointerstitial cells. Co -RNAscope for Cx43 mRNA (red) and CD31 mRNA (green) and nuclear DAPI staining (white) on a normal mouse kidney section in cortical (A), cortical-medullary (B) and medullary (C) areas; size bars 50 μm; Cx43 hybridization signal seems to increase slightly from cortex to the medulla; Note some overlap of Cx43 mRNA and CD31 mRNA expression as indicated by the yellow color merge. (PNG 1974 kb)

High Resolution Image (TIF 1071 kb)

Figure 5 Cx43 mRNA and CX3CR1 mRNA expression in medullary area of a normal mouse kidney section. RNAscope for Cx43mRNA (red) and for CX3CR1 mRNA (green), nuclear DAPI staining (white); size bar 50 μm (PNG 1737 kb)

High Resolution Image (TIF 900 kb)
