# Supplementary figures and images for: Connexin mRNA distribution in adult mouse kidneys
Source: Pflugers Arch. 2021 Aug 7;473(11):1737–47. doi: 10.1007/s00424-021-02608-0 (PMC8528753; doi:10.1007/s00424-021-02608-0)

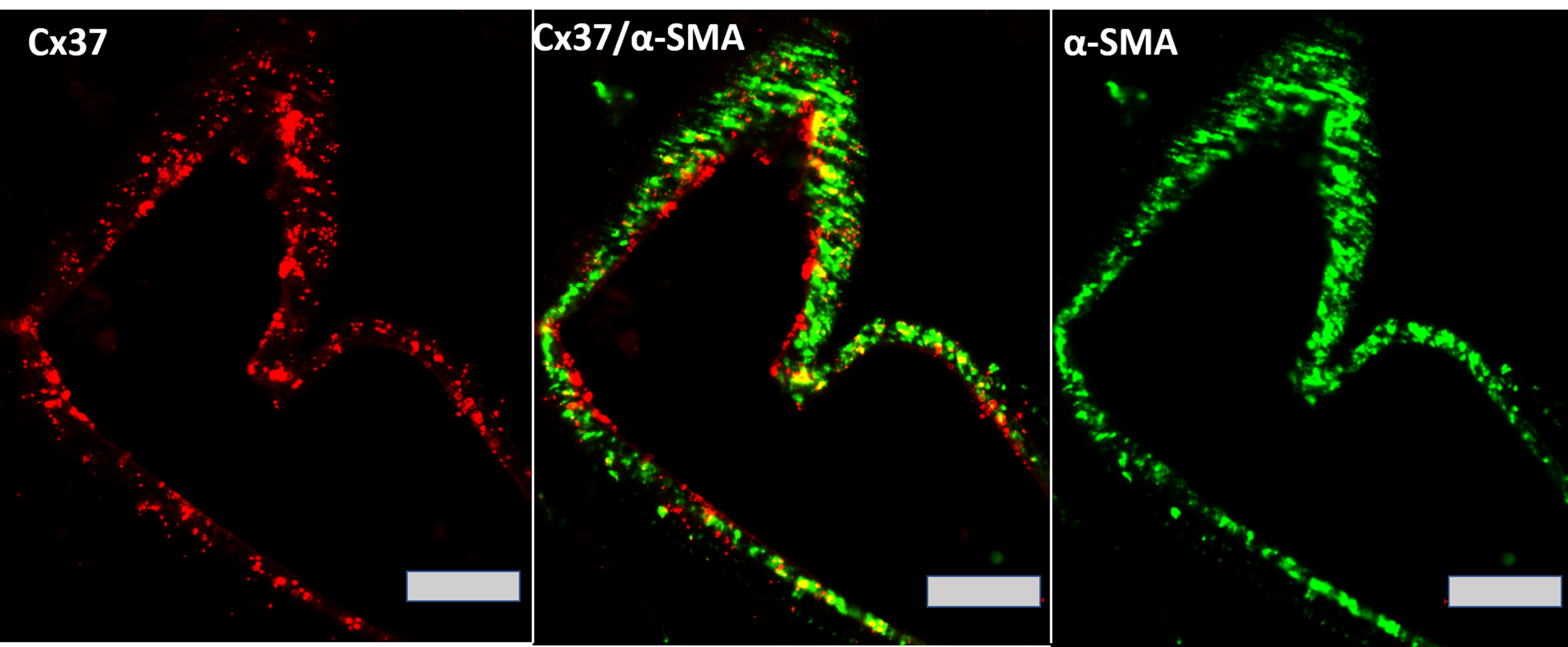

Supplement: Supplementary file 1 — RNA scope showing co-localizition of both Cx37 mRNA (red) and a-SMA mRNA (green) in arterial smooth muscle layer; size bar 50 μm (PNG 1568 kb) [file 424_2021_2608_Fig10_ESM.png]

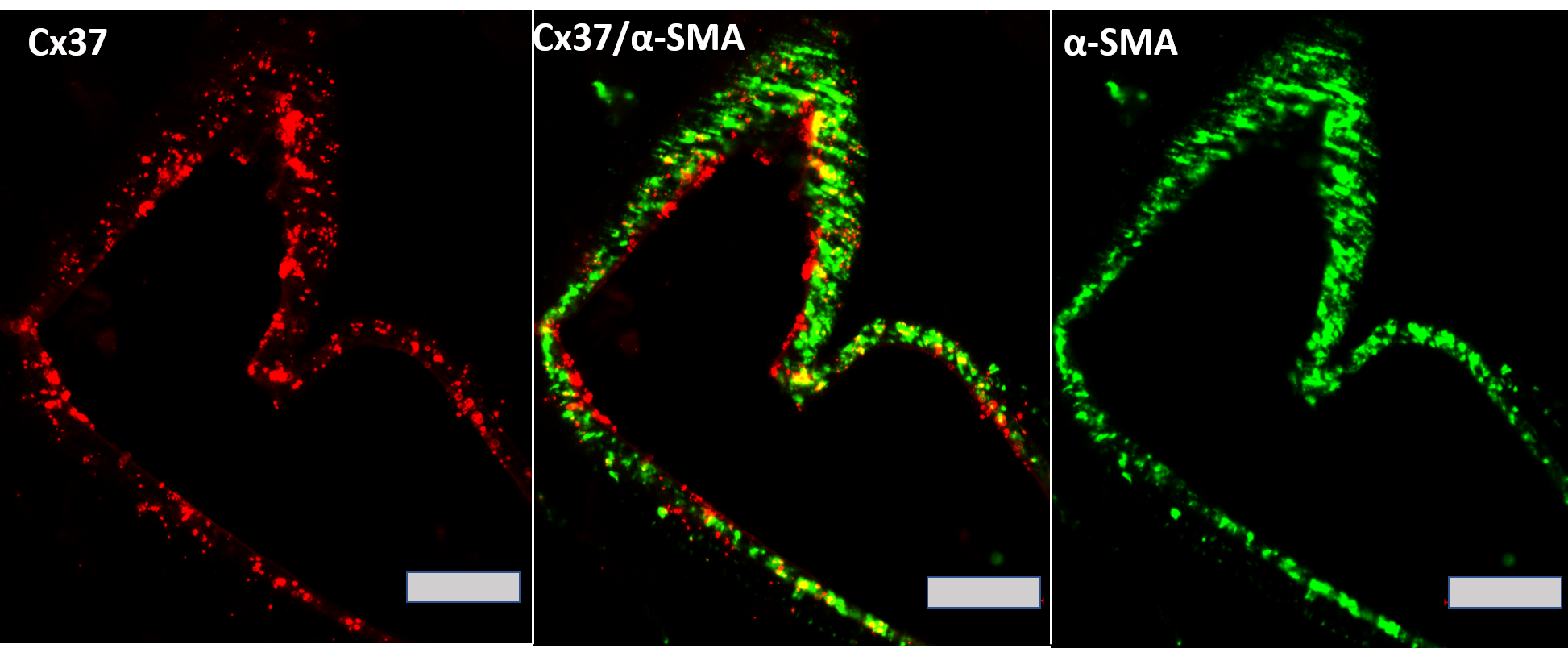

Supplement: Supplementary file 2 — High Resolution Image (TIF 891 kb) [file 424_2021_2608_MOESM1_ESM.tif]

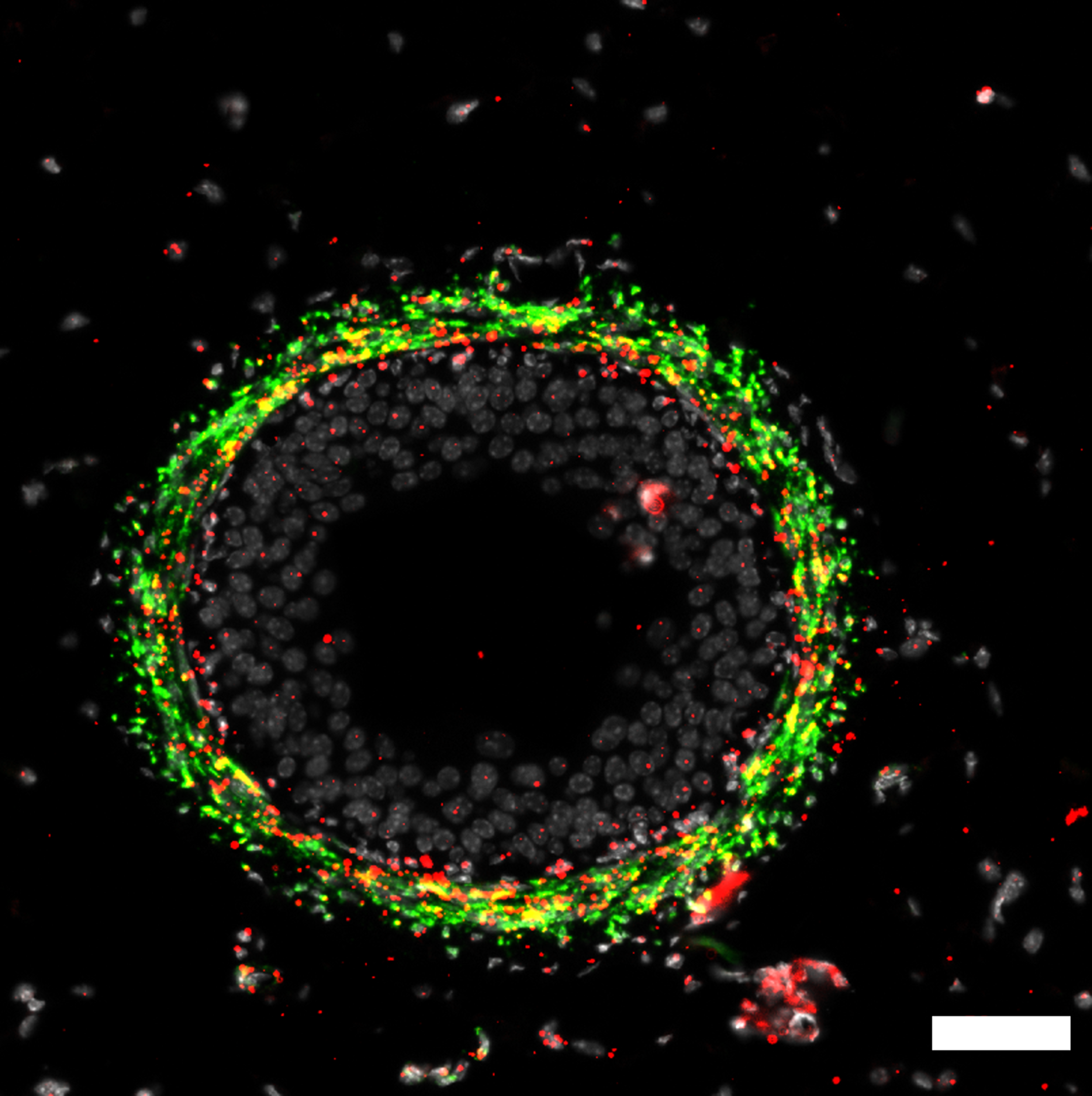

Supplement: Supplementary file 3 — Co RNAscope for Cx45 mRNA (red) and α-SMA (green and nuclear DAPI staining (white) on a cross section of the ureter; size bar 50 (PNG 1952 kb) [file 424_2021_2608_Fig11_ESM.png]

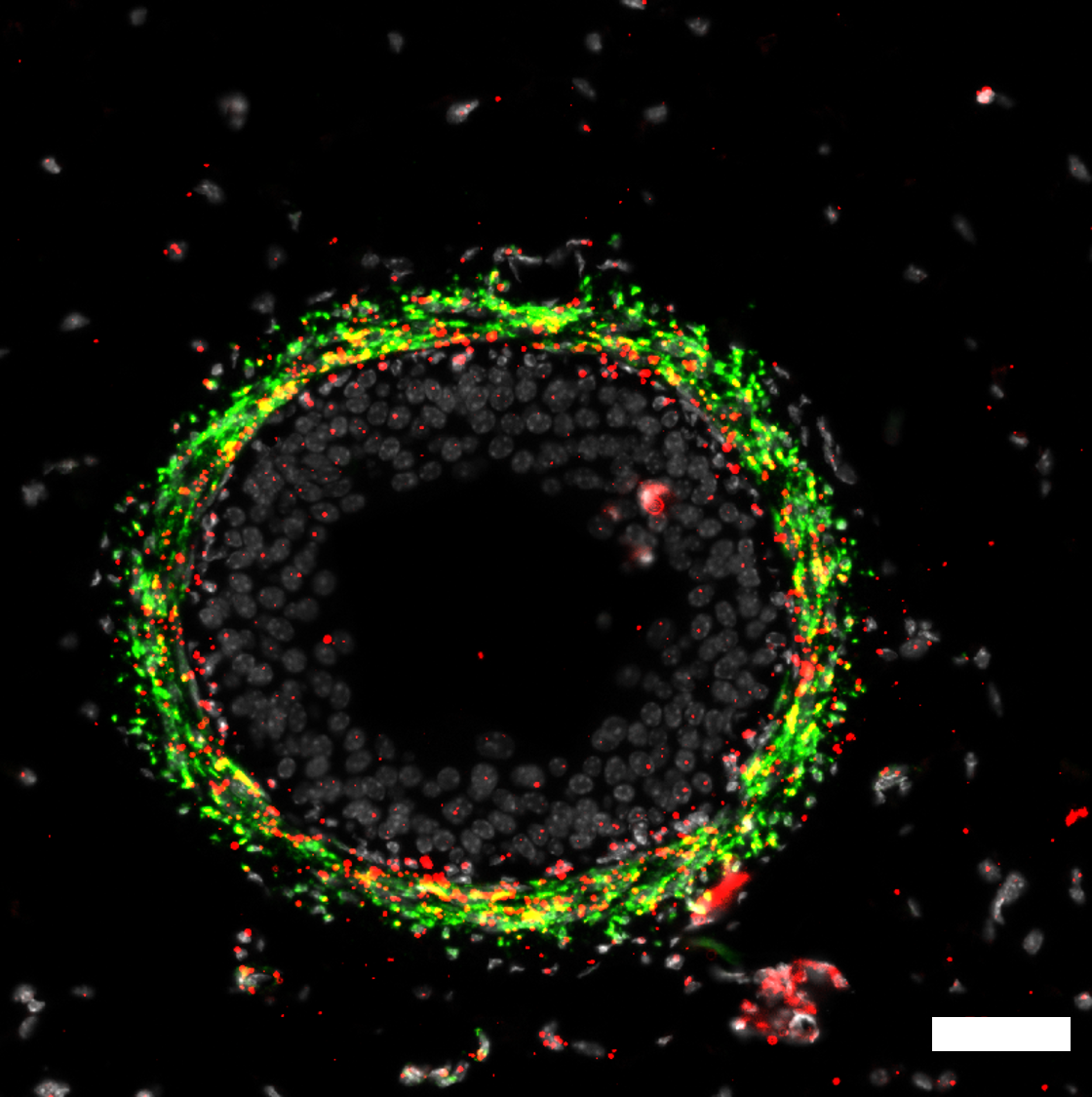

Supplement: Supplementary file 4 — High Resolution Image (TIF 1151 kb) [file 424_2021_2608_MOESM2_ESM.tif]

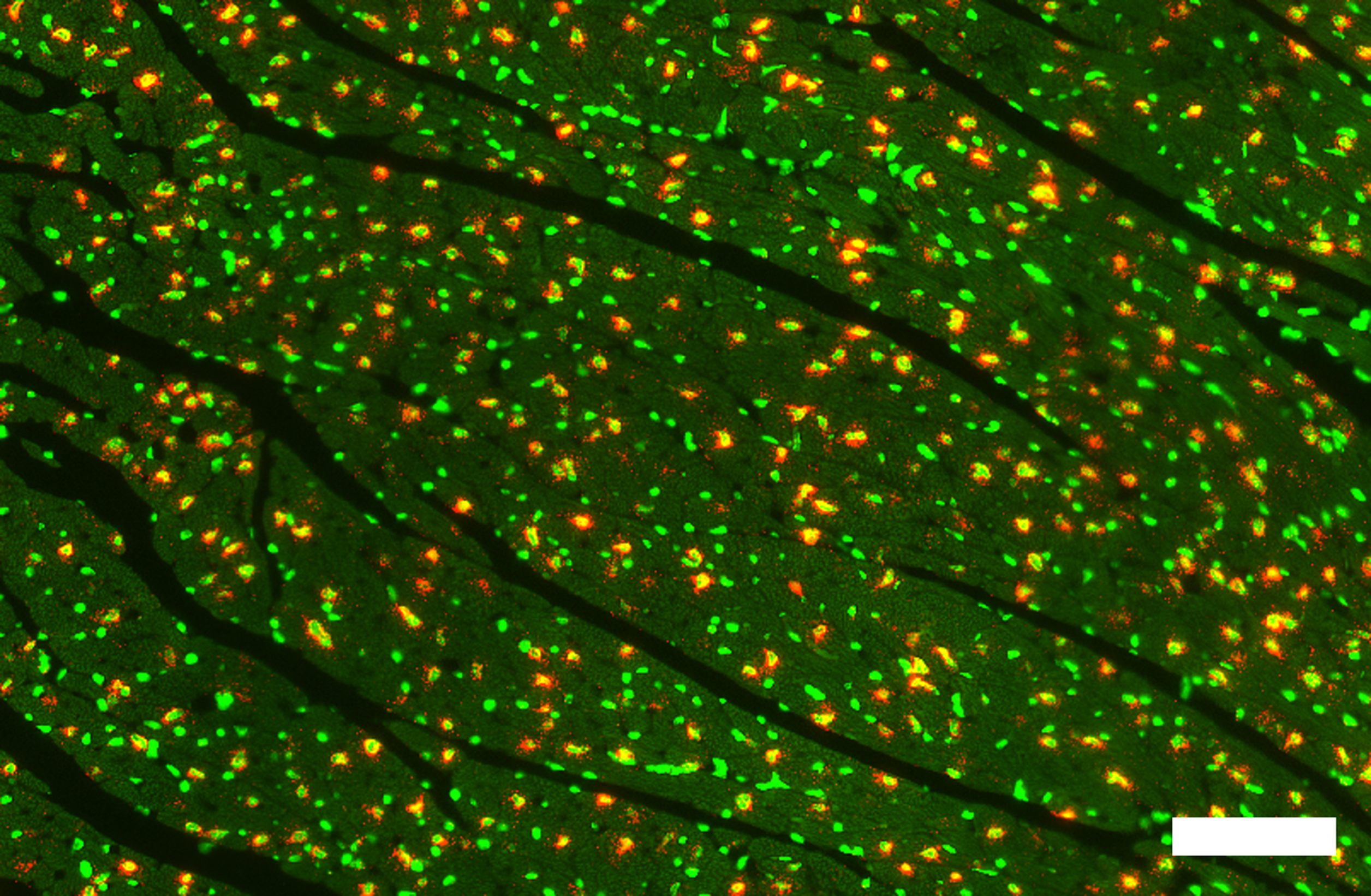

Supplement: Supplementary file 5 — RNAscope for Cx43 mRNA (red) and nuclear staining (green) on a normal adult mouse heart section; size bar 100 μm; Note co-localization of Cx43 mRNA with nuclei as indicated by yellow color merge (PNG 3970 kb) [file 424_2021_2608_Fig12_ESM.png]

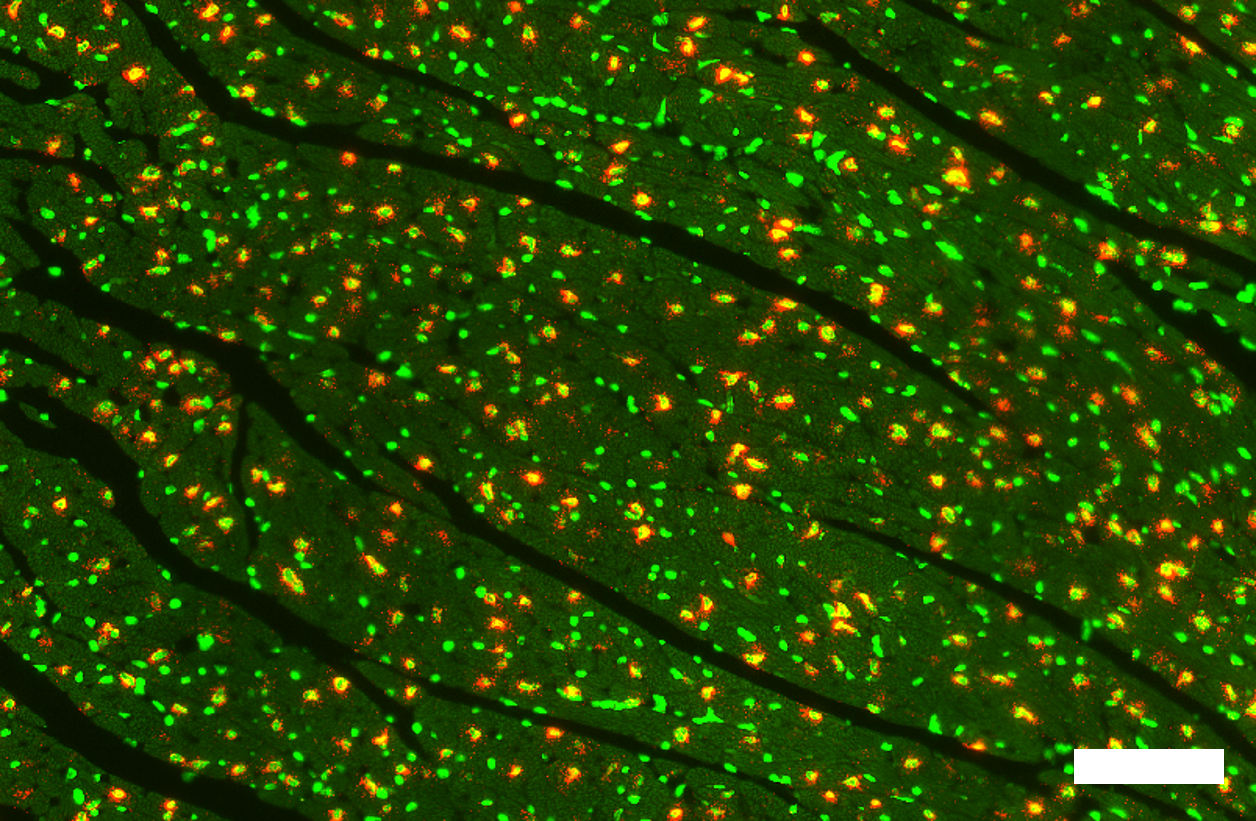

Supplement: Supplementary file 6 — High Resolution Image (TIF 1864 kb) [file 424_2021_2608_MOESM3_ESM.tif]

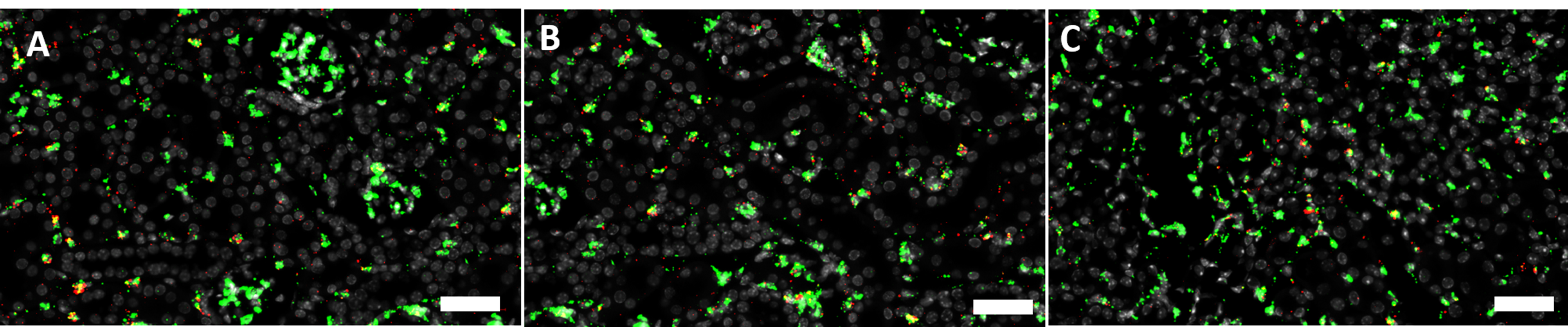

Supplement: Supplementary file 7 — Cx43 mRNA in tubulointerstitial cells. Co -RNAscope for Cx43 mRNA (red) and CD31 mRNA (green) and nuclear DAPI staining (white) on a normal mouse kidney section in cortical (A), cortical-medullary (B) and medullary (C) areas; size bars 50 μm; Cx43 hybridization signal seems to increase slightly from cortex to the medulla; Note some overlap of Cx43 mRNA and CD31 mRNA expression as indicated by the yellow color merge. (PNG 1974 kb) [file 424_2021_2608_Fig13_ESM.png]

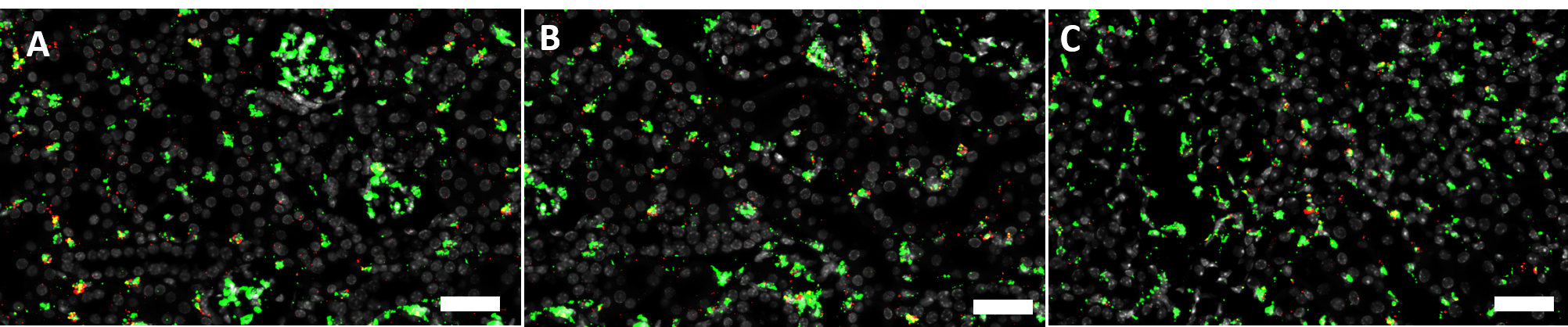

Supplement: Supplementary file 8 — High Resolution Image (TIF 1071 kb) [file 424_2021_2608_MOESM4_ESM.tif]

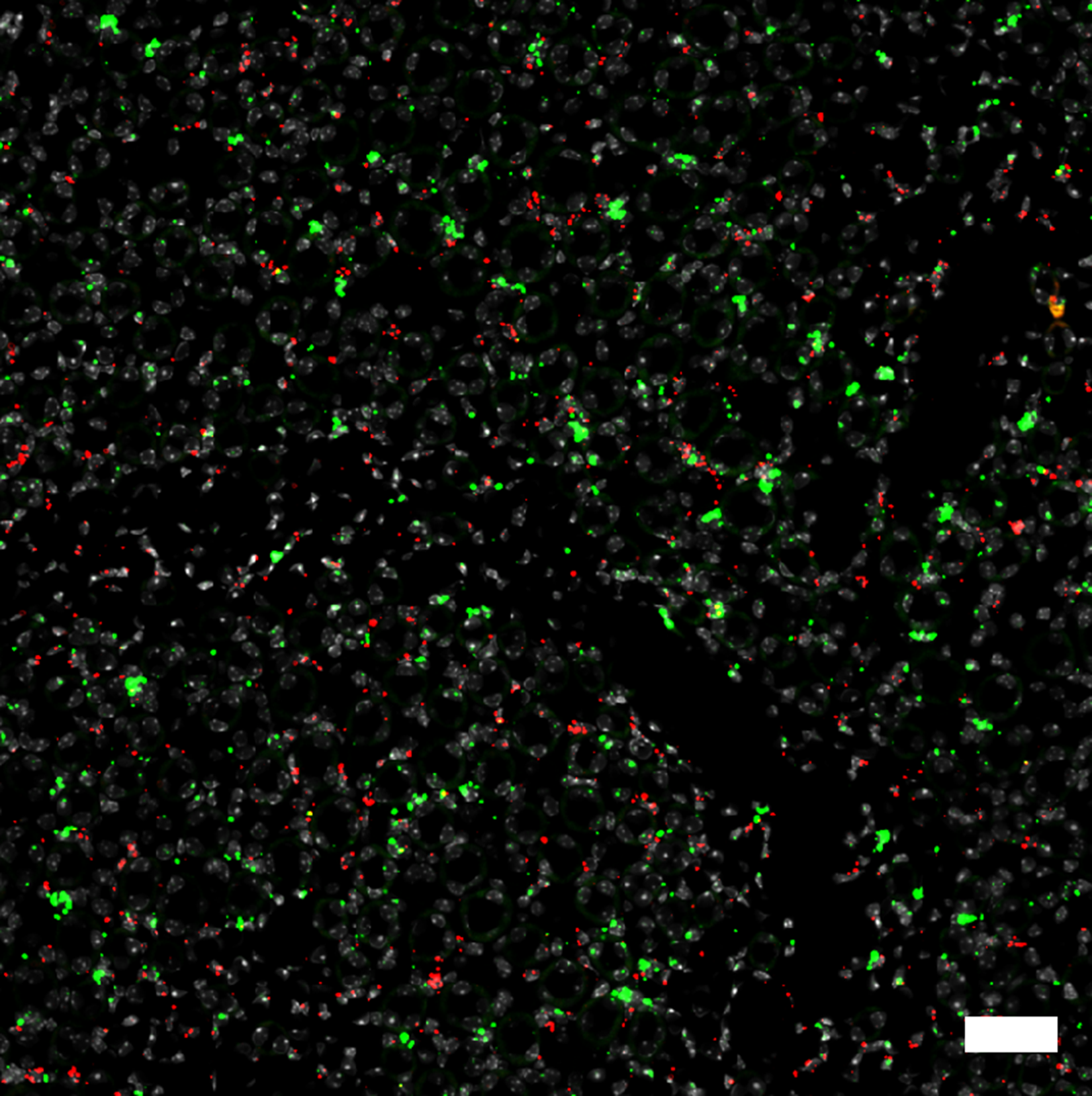

Supplement: Supplementary file 9 — Cx43 mRNA and CX3CR1 mRNA expression in medullary area of a normal mouse kidney section. RNAscope for Cx43mRNA (red) and for CX3CR1 mRNA (green), nuclear DAPI staining (white); size bar 50 μm (PNG 1737 kb) [file 424_2021_2608_Fig14_ESM.png]

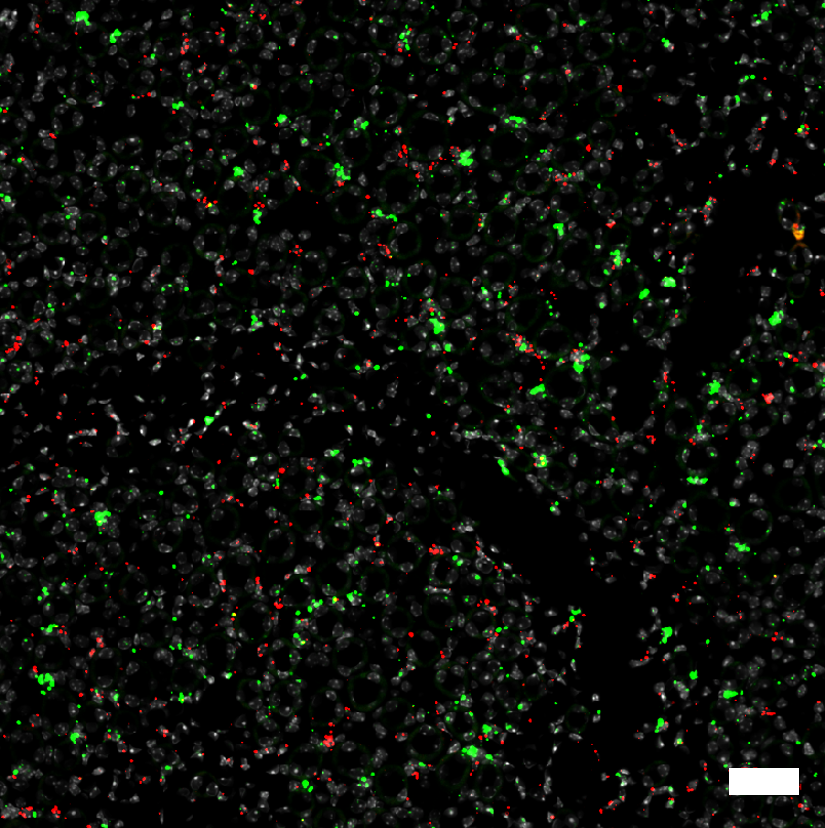

Supplement: Supplementary file 10 — High Resolution Image (TIF 900 kb) [file 424_2021_2608_MOESM5_ESM.tif]
